# Disruption of hematopoiesis attenuates the osteogenic differentiation capacity of bone marrow stromal cells

**DOI:** 10.1186/s13287-022-02708-3

**Published:** 2022-01-24

**Authors:** Changzhen Wang, Hongmei Ning, Jiao Gao, Teng Xue, Ming Zhao, Xiaoxia Jiang, Xiaoming Zhu, Ximin Guo, Hong Li, Xiaoyan Wang

**Affiliations:** 1 The Brain Science Center, Beijing Institute of Basic Medical Sciences, Beijing, 100850 China; 2 Laboratory of Bioelectromagnetics, Beijing Institute of Radiation and Medicine, 27 Taiping Road, Haidian District, Beijing, 100850 China; 3 Department of Hematology, Fifth Medical Center of Chinese PLA General Hospital, Beijing, 100071 China; 4 The Chinese People’s Liberation Army Strategic Support Force Characteristic Medical Center, Beijing, 100101 China

**Keywords:** Mesenchymal stem cells, Hematopoietic cells, Niche, Differentiation

## Abstract

**Background:**

The homeostasis of mesenchymal stem cells (MSCs) is modulated by both their own intracellular molecules and extracellular milieu signals. Hematopoiesis in the bone marrow is maintained by niche cells, including MSCs, and it is indispensable for life. The role of MSCs in maintaining hematopoietic homeostasis has been fully elucidated. However, little is known about the mechanism by which hematopoietic cells reciprocally regulate niche cells. The present study aimed to explore the close relationship between MSCs and hematopoietic cells, which may be exploited for the development of new therapeutic strategies for related diseases.

**Methods:**

In this study, we isolated cells from the offspring of Tie2Cre + and Pten^flox/flox^ mice. After cell isolation and culture, we investigated the effect of hematopoietic cells on MSCs using various methods, including flow cytometry, adipogenic and osteogenic differentiation analyses, quantitative PCR, western bloting, and microCT analysis.

**Results:**

Our results showed that when the phosphatase and tensin homolog deleted on chromosome 10 (Pten) gene was half-deleted in hematopoietic cells, hematopoiesis and osteogenesis were normal in young mice; the frequency of erythroid progenitor cells in the bone marrow gradually decreased and osteogenesis in the femoral epiphysis weakened as the mice grew. The heterozygous loss of Pten in hematopoietic cells leads to the attenuation of osteogenic differentiation and enhanced adipogenic differentiation of MSCs in vitro. Co-culture with normal hematopoietic cells rescued the abnormal differentiation of MSCs, and in contrast, MSCs co-cultured with heterozygous null Pten hematopoietic cells showed abnormal differentiation activity. Co-culture with erythroid progenitor cells also revealed them to play an important role in MSC differentiation.

**Conclusion:**

Our data suggest that hematopoietic cells function as niche cells of MSCs to balance the differentiation activity of MSCs and may ultimately affect bone development.

## Introduction

Mesenchymal stem cells (MSCs) were first found in adult bone marrow and were defined as multipotent stem cells that can functionally differentiate into bone, fat, and cartilage [1–3].The properties of easy amplification and differentiation render them an intriguing cell source for regenerative medicine [2–4]. Both the intracellular and extracellular mechanisms that regulate the differentiation and stemness of MSCs have been studied to a certain extent [5–9].

In the bone marrow niche, MSCs are usually viewed as supporting cells for hematopoiesis, and their essential ability to regulate hematopoiesis has been comprehensively explored [10–13]. It has been shown that hematopoietic stem/progenitor cells (HSPCs) can regulate the regeneration of niches that maintain them [14], which suggests that signaling between different cells in the bone marrow niche is reciprocal. However, knowledge of how hematopoietic cells reversely affect MSC homeostasis remains unclear. Recent findings suggest that MSCs and HSPCs reside in immediate proximity and function in cooperation with each other [15]. It has been verified that bone marrow HSPCs can guide MSCs to differentiate toward the osteoblastic lineage in vitro and in vivo [16, 17]. Mononuclear phagocytes in the vicinity of Nestin^+^ MSCs promote the retention of HSPCs in the bone marrow by regulating the secretion of related cytokines in these MSCs [18]. These results suggest that both hematopoietic cells and MSCs are constituents of the bone marrow entity; they participate in maintaining the stability of the bone marrow niche through reciprocal regulation. A better understanding of the microenvironment of MSCs can offer new avenues for regenerative medicine and might provide insights into new therapeutic strategies for hematopoietic system diseases.

Phosphatase and tensin homolog deleted on chromosome 10 (Pten) is a potent tumor suppressor gene, and its loss in HSPCs leads to leukemogenesis [19–21]. In this study, using cells isolated from the offspring of Tie2Cre^+^ and Pten^flox /flox^ mice, we investigated the effect of hematopoietic cells on MSCs. Although the appearance of Cre^+^Pten^+/flox^ mice is normal, haploinsufficiency of the Pten gene in hematopoietic cells leads to gradually attenuated erythropoiesis and osteogenesis. MSCs isolated from Cre^+^Pten^+/flox^ mice express Pten normally, but they deposit less extracellular calcium when induced to differentiate into osteoblasts in vitro. Simultaneously, Cre^+^Pten^+/flox^ MSCs produced many more adipocytes in adipogenic culture. Furthermore, the abnormal differentiation of MSCs from CrePten^+/flox^ mice can be rescued by co-culture with normal bone marrow hematopoietic cells. Based on these data, we propose that hematopoietic cells function as niche cells for MSCs.

## Materials and methods

### Mice

Female Tie2Cre^±^ mice and male Pten^flox/flox^ mice were purchased from Jackson Laboratory (Bar Harbor, MA, USA). The mice were bred and maintained in the animal laboratory of the Beijing Institute of Basic Medical Sciences (Beijing, China). The F1 generation mice for the experiments were obtained by hybridization of the two kinds of mice. All mouse experiments complied with all relevant ethical regulations and were performed according to protocols approved by t the Institutional Animal Care and Use Committee of National Beijing Center for Drug Safety Evaluation and Research.

### Cell isolation and culture

The F1 offspring mice at various ages were sacrificed by CO_2_ suffocation followed by cervical dislocation. Cells were then isolated and cultured as previously described [22] with modifications. Briefly, femurs and tibias were separated and cut into small pieces after the soft tissues were cleared. Both bone and marrow cells were cultured in α-modified Eagle medium with 10% fetal bovine serum (FBS, Excell), 100 U/mL penicillin, 100 μg/mL streptomycin and 50 μM 2-mercaptoethanol. The plates were then placed in a humidified incubator at 37 °C and 5% CO_2_ and maintained for at least 48 h. Half the medium was refreshed after three days of culture. When approximately 70–80% confluence was reached, the bone chips and supernatant cells were gently removed, and the adherent cells were digested with 0.25% trypsin/EDTA for further expansion. For the colony-forming unit of fibroblast (CFU-f) assay, bone chip pieces and cells were washed three times with medium and filtered through a nylon screen (40 μm) (BD, NJ, USA), and plated at the density of 10^6^ cell/well in 6-well plates for 2 weeks. After that, the cells were stained with 0.5% crystal violet, and colonies were scored. For flow cytometry analysis, the cells were transferred to red blood cell lysis buffer (Biolegend, San Diego, USA) and incubated for 10 min. After washing, the cells were filtered through a nylon screen, counted and stained with antibodies for flow cytometry.

### Flow cytometry

For flow cytometry analysis, adherent cells at passage 3–4 were harvested using 0.25% trypsin/EDTA and stained for 30 min at 4 °C with phycoerythrin (PE)-conjugated anti-mouse monoclonal antibodies. Antibodies recognizing CD29, CD31, and CD45 were purchased from eBioscience (San Diego, CA, USA). Antibodies against CD44, CD140a, and CD140b were purchased from BioLegend. Fluorescein isothiocyanate (FITC) conjugated Ter119 and CD45 used to stain single bone marrow cells were purchased from Biolegend. The stained cells were analyzed using a FACSCalibur flow cytometer (BD, NY, USA).

### Adipogenic and osteogenic differentiation assays

For osteogenic differentiation, cells were incubated in Dulbecco’s modified Eagle’s medium (DMEM) with 10% FBS supplemented with 0.2 mM ascorbic acid, 10^–7^ M dexamethasone, and 10 mM glycerol phosphate (Sigma-Aldrich, MO, USA) for 3 weeks, and the mineralization capacity was evaluated by von Kossa staining. Calcium deposition was quantified according to the instructions of the calcium colorimetric assay kit (BioVision, San Francisco, CA, USA). For adipogenic differentiation, the cultures were incubated in DMEM supplemented with 10% FBS, 10^–6^ M dexamethasone, 0.5 μM isobutyl-methylxanthine, 10 ng/mL insulin, and 60 μM indomethacin (Sigma-Aldrich, MO, USA) for 2 weeks. Cells were fixed with paraformaldehyde and stained with fresh Oil Red O solution (Sigma-Aldrich, MO, USA).

### Co-cultures

MSCs at passage 4 were seeded at a density of 3000 cells/cm^2^ in a 24-well plate. After 1 d in culture, 2 × 10^6^ marrow cells from either Pten^+/flox^ or Cre^+^Pten^+/flox^ animals were added to the cultures. The co-cultures were carried out in Iscove’s modified Dulbecco’s medium containing 15% FBS, 100 U/mL penicillin, 100 μg/mL streptomycin, and 50 μM 2-mercaptoethanol. After three days of co-culture, the supernatant cells were removed gently, and the cultures were then transferred to medium favoring osteogenic or adipogenic differentiation.

Transwell co-culture was performed in 24-well plates, with the MSCs plated in the lower well, and the bone marrow cells plated in the upper well. The upper wells with marrow cells were discarded after three days of co-culture, and the medium in the lower well was changed to osteogenic or adipogenic differentiation medium.

To evaluate erythroid progenitor co-culture, whole bone marrow was treated with red blood cell lysis reagent and nucleated Ter119^+^ cells were harvested as per the instructions of the isolation kit (Militenyi Biotec, Bergisch Gladbach, Germany). Approximately 3 × 10^6^ Ter119^+^ cells were then incubated with MSCs in 24-well plates for three days, after which the differentiation assays were performed.

### Quantitative PCR (qPCR)

Total RNA was extracted from cells using TRIzol (Invitrogen, CA, USA) and reverse transcription was performed with Reverse Transcriptase XL (TaKaRa, Tokyo, Japan). To measure OPN, OCN, FAB4, and PPARγ2 mRNA expression in the differentiation-induced cells and gene expression (including OPG, OSM, CCL3, and TNF-α) in bone marrow cells, the SYBR assay kit was used (ABI, Foster, USA). The corresponding primers were designed and synthesized by Shenggong Bioengineering Co., Ltd. (Beijing, China; Table 1). Briefly, 1 μL cDNA was mixed with 7.5 μL SYBR Green PCR Master Mix and 0.2 μL of primers. Samples were then added to 6.30 μL of water (for a final volume of 15 μL). qPCR was carried out according to the following conditions: 95 °C for 10 min, 40 × (95 °C for 15 s and 60 °C for 1 min), and 95 °C for 15 min, 60 °C for 60 min, 95 °C for 15 min. All reactions were run in triplicate on an ABI StepOnePlus Real-Time PCR System (Foster, USA).

**Table 1:** Primers used for qRT-PCR

Genes	Forward	Reverse
OPG	5’ CAGAGAAGCCACGCAAAAGTG 3’	5’ AGCTGTGTCTCCGTTTTATCCT 3’
TNF α	5’ ATGTCGGCTCCAGGACCTTA 3’	5’ GGTAGTAACTGTTGACACCCACT 3’
OSM	5’ TCCGCCTCCAAAACCTGAAC 3’	5’ TTATGCCGAGGATATTGTGCC 3’
CCL3	5’ TGTACCATGACACTCTGCAAC 3’	5’ CAACGATGAATTGGCGTGGAA 3’
OPN	5’ AGCAAGAAACTCTTCCAAGCAA 3’	5’ GTGAGATTCGTCAGATTCATCCG 3’
OCN	5’ GGGCAATAAGGTAGTGAACAG 3’	5’ GCAGCACAGGTCCTAAATAGT 3’
PPARγ2	5’ TTTTCCGAAGAACCATCCGATT 3’	5’ ATGGCATTGTGAGACATCCCC 3’
FAB4	5’ TACATGGCTTCCGTGCAAGTG 3’	5’ CACAGAGTCGTCATCCGTCA 3’
actin	5’ GGCCCAGAGCAAGAGAGGTA 3’	5’ CATGTCGTCCCAGTTGGTAACA 3’

*OPG* osteoprotegerin, *OSM* oncostatin M, *TNF α* tumor necrosis factor alpha, *CCL* chemokine (C–C motif) ligand, *OPN* osteopontin, *OCN* osteocalcin, *PPARγ* peroxisome proliferators-activated receptor –gamma, *FAB4* fatty acid-binding protein 4

### Western blot analysis

Proteins were collected and detected by western blot analysis as previously reported [23]. Anti-Pten and anti-actin antibodies were purchased from Cell Signaling Technology (Danvers, MA, USA).

### MicroCT analysis

The right femur of each mouse was fixed in 10% formalin for 48 h and then stored in 70% ethanol at 4 °C. MicroCT analysis was performed using a Skyscan1076 (Bruker, Karlsruhe, Germany). The trabecular bone microarchitecture in the distal femoral metaphysis and cortical bone at the femoral midshaft were determined. Cancellous bone was assessed in the region starting 120 μm proximal to the growth plate and extending by 464 transverse slices. Bone volume fraction (BV/TV), trabecular number (Tb.N, /mm), trabecular thickness (Tb.Th, mm), and trabecular separation (Tb.Sp, mm) were analyzed.

### Phase contrast imaging

Images were collected using an Olympus CK2 inverted microscope (Tokyo, Japan) with a 10/0.25 NA A10PL objective and were acquired using a Nikon Coolpix 995 camera (Tokyo, Japan) and a Nikon TE300 inverted microscope (Tokyo, Japan) with a Nikon 20/0.45 NA Plan Composite images were assembled.

### Statistical analysis

For quantitative assays, data are reported as the mean ± SEM and compared using an unpaired Student’s *t*-*test*. Statistical significance was set at *P* ≤ 0.05.

## Results

### Heterozygous loss of Pten gradually leads to abnormal marrow erythropoiesis

**Fig. 1:**
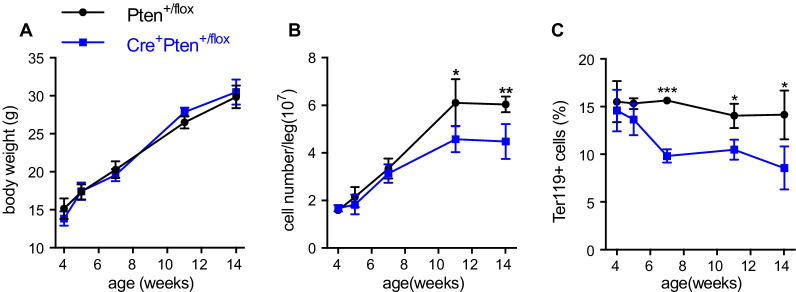
Development of Pten^+/flox^ and Cre^+^Pten^+/flox^ mice. **A** The body weight of Pten^+/flox^ and Cre^+^Pten^+/flox^ mice over time. **B** Bone marrow cellularity in the femur and tibia with the growth of Pten^+/flox^ and Cre^+^Pten^+/flox^ mice. **C** The frequency of the Ter119^+^ subpopulation cells in Pten^+/flox^ and Cre^+^Pten^+/flox^ mice at various ages. Data are presented as mean ± SEM. *P < 0.05, **P < 0.01 and ***P < 0.001

The offspring mice were generated by mating Pten^flox/flox^ mice with Tie2Cre^±^ transgenic mice. Although it has been reported that subtle variations in Pten dosage can determine cancer susceptibility [23, 24], no apparent difference was found between Pten^+/flox^ mice and Cre^+^Pten^+/flox^ mice (Fig. 1A and data not shown). The bone marrow of mice at various ages was flushed out, and hematopoietic subsets were analyzed by flow cytometry. With growth, the increase in bone marrow cellularity in Cre^+^Pten^+/ flox^ mice slowed down slightly compared to that in Pten^+/flox^ mice (Fig. 1B). Flow cytometry results showed that although the frequency of nucleated Ter119^+^ erythroid cells was normal in young mice, it gradually became lower in Cre^+^Pten^+/ flox^ mice compared to Pten^+/flox^ mice (Fig. 1C). These data indicate that marrow erythropoiesis in the Cre^+^Pten^+/flox^ mouse bone marrow progressively increased with time.

### Heterozygous loss of Pten alters the differentiation activity of mesenchymal stem cells

**Fig. 2:**
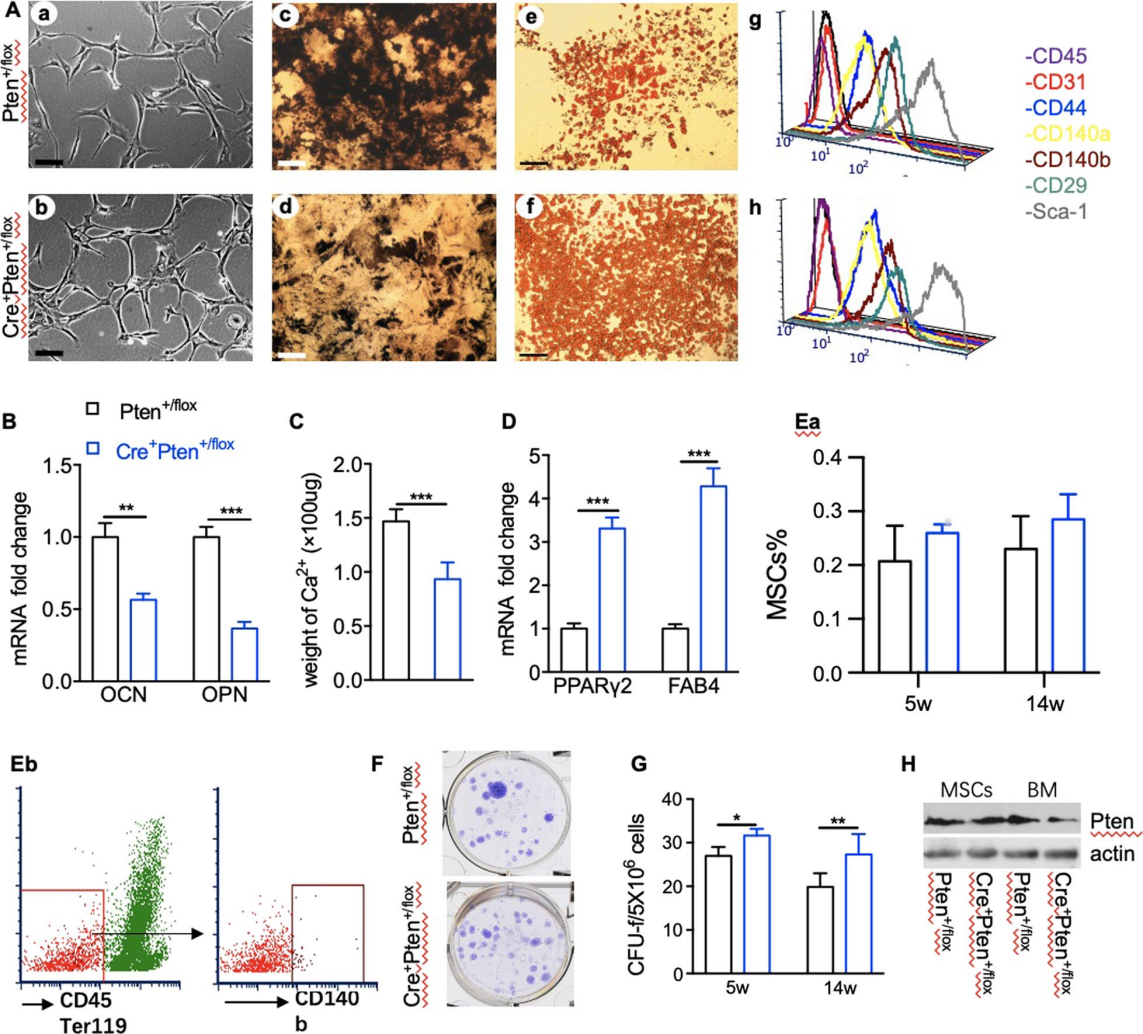
Characteristics of Pten^+/flox^ and Cre^+^Pten^+/flox^ MSCs. **Aa**, **b** Representative morphologies of the adherent cells derived from Pten^+/flox^ and Cre^+^Pten^+/flox^ mice are shown at passage 3. **Ac**, **d** von Kossa staining shows the mineral deposition of the differentiated Pten^+/flox^ and Cre^+^Pten^+/flox^ MSCs. **Ae**, **f** Oil Red O staining shows the induced adipocytes from Pten^+/flox^ and Cre^+^Pten^+/flox^ MSCs. **Ag**, **h** Flow cytometric analysis shows the uniform and identical immunophenotypes of Pten^+/flox^ and Cre^+^Pten^+/flox^ cells at passage 3. **B** Relative level of OCN and OPN mRNA in Pten^+/flox^ and Cre^+^ Pten^+/flox^ cells cultured in osteo-induced medium. **C** Quantity of calcium deposition of MSCs with different genotypes during osteoblastic differentiation. **D** The mRNA expression of genes specific for adipo-induction. **Ea**–**b** The frequency of MSCs in bone marrow was approximated using the markers Ter119^−^CD45^−^CD140b^+^. **F**, **G** CFU-f assay shows Cre^+^Pten^+/flox^ marrow cells possess higher colony-forming efficiency. **H** Western blot shows the expression of Pten in different cells. Data are presented as mean ± SEM. *P < 0.05, **P < 0.01 and ***P < 0.001

To test whether the characteristics of bone marrow MSCs in Cre^+^Pten^+/flox^ mice were altered, MSCs of femurs and tibias at various ages were isolated, cultured, and subjected to differentiation tests. At all ages examined, no difference was found in the morphology of the in vitro cultured cells (Fig. 2Aa, b). Flow cytometry analysis also demonstrated no abnormalities in the surface markers that characterize MSCs (Fig. 2Ag, h). Cells of both genotypes uniformly expressed CD44, CD140a, CD140b, CD29, and Sca-1, and were negative for the hematopoietic marker CD45 and endothelial marker CD31.

Cells with homogenous morphology and phenotype at passage 3 to 4 were replated in conditions known to induce the differentiation of MSCs into osteogenic or adipogenic lineages. Compared to MSC from Pten^+/flox^ mice, MSCs from Cre^+^Pten^+/flox^ mice showed much less mineral accumulation in osteo-inductive medium (Fig. 2Ac, d). The mRNA expression of genes related to osteoblastic differentiation was also significantly reduced (Fig. 2B). The calcium content in the extracellular matrix was quantified, and the results showed that the calcium deposition of MSCs of the Cre^+^Pten^+/flox^ genotype decreased by nearly half (Fig. 2C). In contrast, after two weeks of culture in medium that favored adipogenic differentiation, more adipocytes appeared in the Cre^+^Pten^+/flox^ MSCs cultures (Fig. 2Ae, f), and the results from qRT-PCR analyses also indicated a substantial increase in mRNA expression of proliferation-activated receptor γ2 (PPARγ2) and fatty acid-binding protein 4 (FAB4), genes specific for adipocytes (Fig. 2D).

The percentage of MSCs in the bone marrow was analyzed using flow cytometry. The results showed that the frequency of MSCs is increased slightly with heterozygous loss of Pten (Fig. 2Ea–b). However, in vitro characterization of this population also showed an obvious increase in the CFU-F number (Fig. 2F, G). To determine whether Pten was deficient in Cre^+^Pten^+/flox^ MSCs and caused abnormal in vitro differentiation, expanded MSCs at passage 3 were subjected to western blotting to check Pten levels. The results showed that although the expression of Pten decreased significantly in whole bone marrow cells from Cre^+^Pten^+/flox^ mice (Fig. 2H), Pten protein levels in Cre^+^Pten^+/flox^ MSCs were comparable to the normal expression Pten^+/flox^ MSCs. These data suggest that the abnormal in vitro differentiation activity of Cre^+^Pten^+/flox^ MSCs may be caused by inappropriate hematopoiesis in the in vivo milieu.

### Heterozygous null Pten leads to attenuated osteogenesis in vivo

**Fig. 3:**
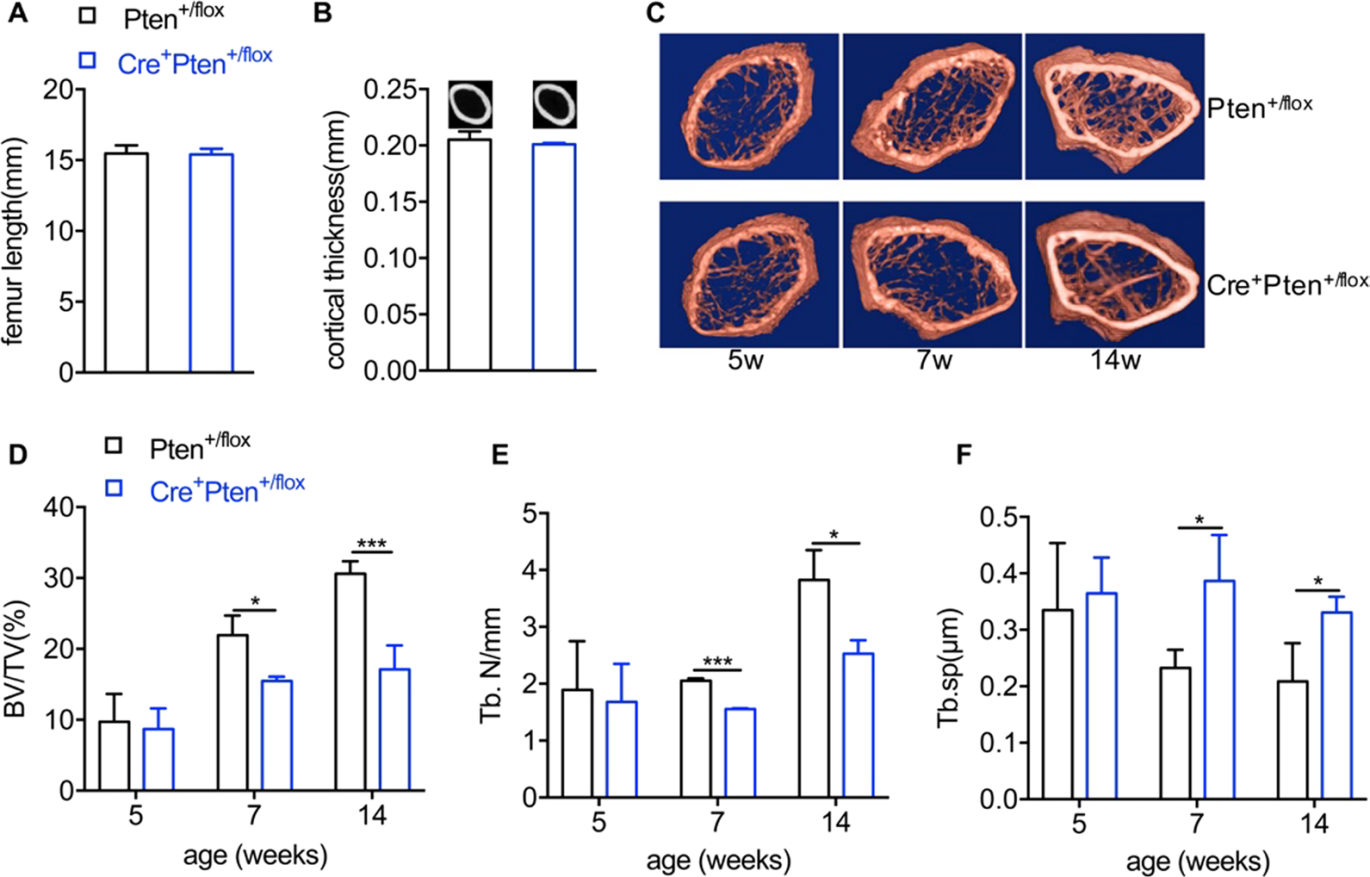
The in vivo bone development of Pten^+/flox^ and Cre^+^Pten^+/flox^ mice. **A** Femur lengths of 14-week-old mice with different genotypes. **B** Femur cortical thicknesses of 14-week-old mice with different genotypes. **C**–**F** MicroCT analysis of proximal femurs of Pten^+/flox^ and Cre^+^Pten^+/flox^ mice at various ages. Data are presented as mean ± SEM. *P < 0.05, **P < 0.01 and ***P < 0.001

Because MSCs and their osteogenic derivatives are responsible for bone formation, to address whether in vivo bone development is affected in Cre^+^Pten^+/flox^ mice, femurs of various ages were isolated, and bone parameters were analyzed. No obvious abnormalities were observed in femur length or cortical thickness in Cre^+^Pten^+/flox^ mice (Fig. 3A, B). However, the microCT results showed retarded cancellous bone formation in the femoral epiphysis of Cre^+^Pten^+/flox^ mice (Fig. 3C–F). In mice younger than 5 weeks, no obvious differences in bone parameters were found between mice with different genotypes. However, after 7 weeks, in Cre^+^Pten^+/flox^ mice, bone mass was markedly decreased compared to that in Pten^+/flox^ mice (Fig. 3D). The trabecular number in the femoral metaphysis of Cre^+^Pten^+/flox^ mice was much lower than that of Pten^+/flox^ mice and the trabecular space increased accordingly (Fig. 3C, E–F). Together, these data indicate that heterozygous loss of Pten caused a gradual decrease in cancellous bone formation.

### Normal hematopoietic progenitor cells can rescue the abnormal osteoblastic differentiation of MSCs in vitro

To investigate whether abnormal hematopoiesis is responsible for the weakened osteogenic and enhanced adipogenic differentiation of the Cre^+^Pten^+/flox^ MSCs, MSCs of different genotypes were co-cultured with bone marrow hematopoietic cells derived from Cre^+^Pten^+/flox^ or Pten^+/flox^ mice for 3 days. The co-culture maintenance medium was then gently replaced with medium to stimulate differentiation.

**Fig. 4:**
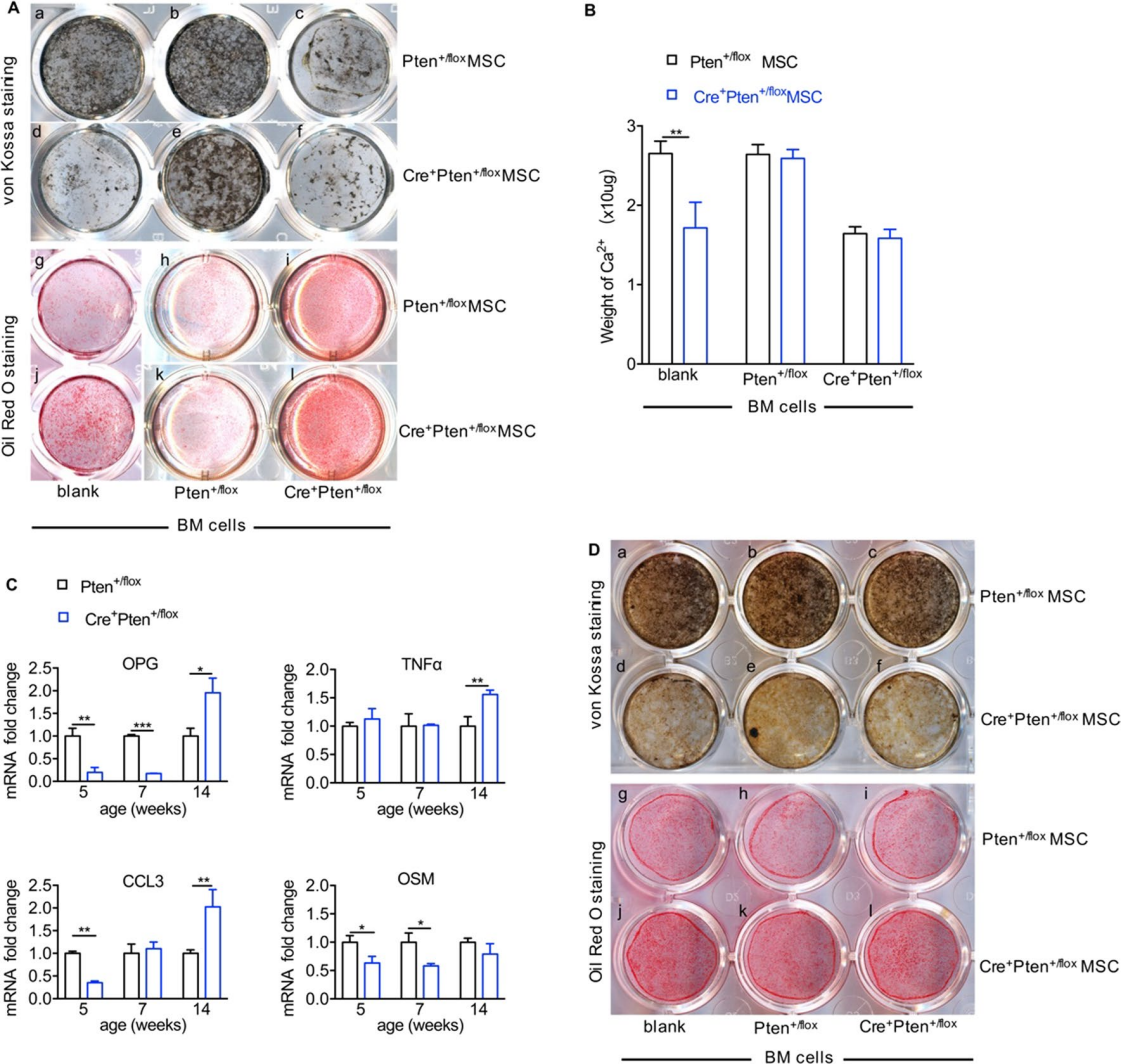
The differentiation of Pten^+/flox^ and Cre^+^Pten^+/flox^ MSCs after cross co-culture with different bone marrow blood cells from 5-week-old mice. **Aa**–**f** von Kossa staining showing the alteration of extracellular mineral deposition of MSCs after incubation with different bone marrow blood cells. **Ag**–**l** Oil Red O staining showing adipocyte formation after incubation with bone marrow blood cells for 3 days and adipo-induction for 10 days. **B** Quantity of calcium deposition of MSCs in osteo-induction medium after incubation with bone marrow blood cells for 3 days. **C** The relative mRNA expression of genes related to osteogenesis in Pten^+/flox^ and Cre^+^Pten^+/flox^ bone marrow cells. **Da**–**f** The osteoblastic differentiation and **Dg**–**l** adipo-differentiation of Pten^+/flox^ and Cre^+^Pten^+/flox^ MSCs when Transwells were inserted into co-culture to block direct contact between blood cells and MSCs. Data are presented as mean ± SEM. *P < 0.05, **P < 0.01 and ***P < 0.001

Surprisingly, when co-cultured with the same hematopoietic cells, MSCs of different genotypes displayed similar differentiation activity regardless of the age of the hematopoietic cells. As shown in Fig. 4A, when co-cultured directly with Pten^+/flox^ bone marrow cells, the impaired extracellular mineral deposition of Cre^+^Pten^+/flox^ MSCs was recovered (Fig. 4Ad, e, B), and the defectively enhanced adipocyte formation was also corrected (Fig. 4Aj, k). Conversely, compared to Pten^+/flox^ blood cells, Cre^+^Pten^+/flox^ blood cells made the co-cultured normal MSCs deposit much less calcium (Fig. 4Aa, c, B) and form more adipocytes (Fig. 4Ag, i). Hematopoietic cells may affect MSC differentiation by secreting soluble factors. qRT-PCR analysis showed that the expression of several candidate factors known to regulate osteoblastic differentiation, such as OPG, CCL3, OSM, and TNFα, were significantly altered in Cre^+^Pten^+/flox^ bone marrow cells (Fig. 4C). Transwell plates were then used to determine the relative contribution of secreted soluble factors to MSC differentiation. Strikingly, the Transwell almost completely blocked the influence of bone marrow blood cells on extracellular mineral deposition (Fig. 4Da–f) and adipocyte differentiation (Fig. 4Dg–l). Collectively, these findings indicate that in the bone marrow, hematopoietic cells modulate the differentiation of MSCs, and the close proximity between blood cells and MSCs is essential for MSC differentiation.

### Erythroid progenitor cells play an important role in balancing MSC differentiation

**Fig. 5:**
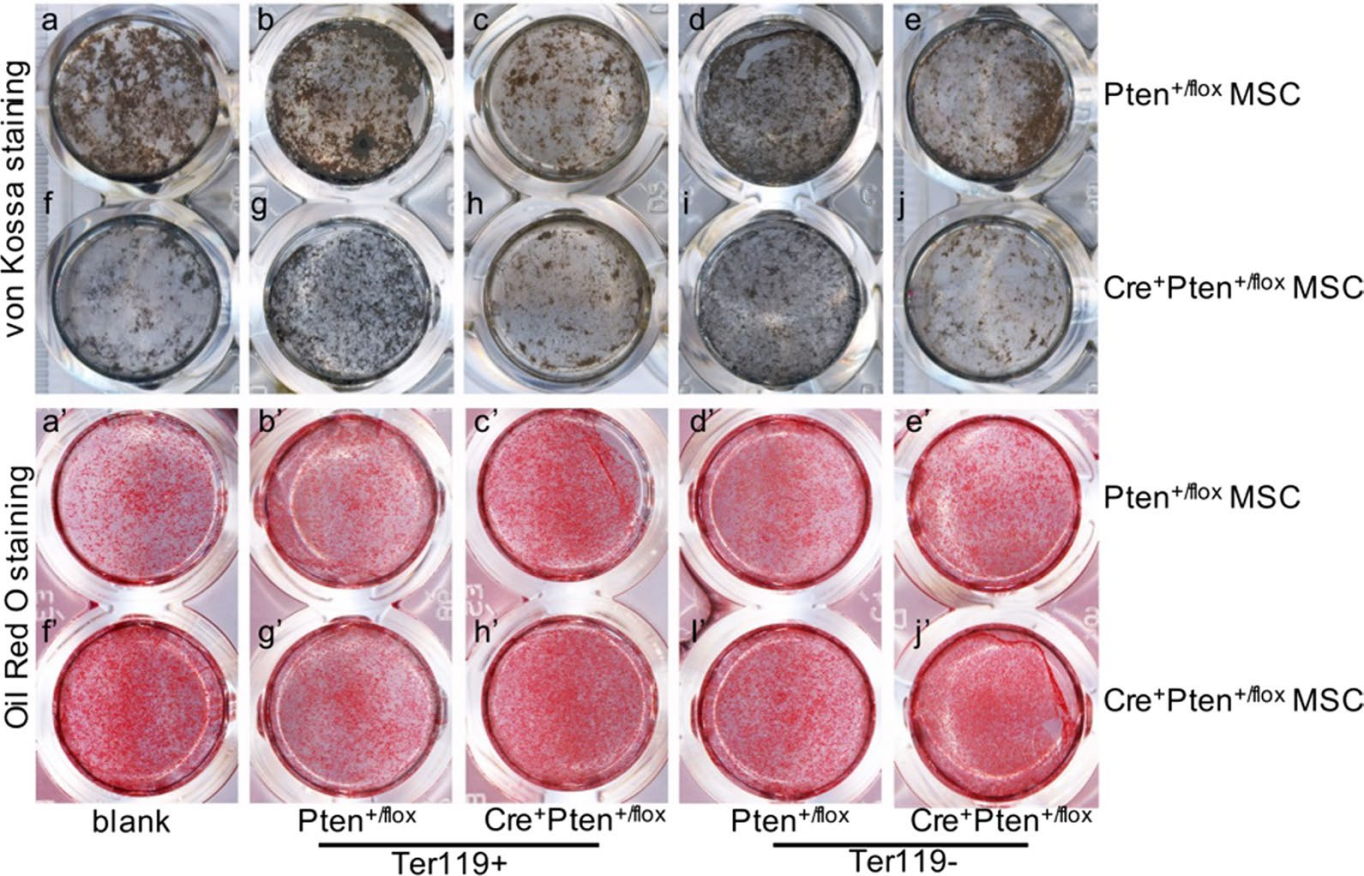
The differentiation of Pten^+/flox^ and Cre^+^Pten^+/flox^ MSCs after cross co-culture with bone marrow Ter119^+^ or Ter119^−^ subpopulations. **a**–**j** von Kossa staining showing the alteration of mineral deposition of MSCs when co-cultured with Ter119^+^ or Ter119^−^ erythroid progenitor cells. **a’**–**j’** Oil Red O staining displaying the influence the Ter119^+^ and Ter119^−^ erythroid progenitor cells on the adipogenic differentiation of the co-cultured MSCs

The relative contribution of erythrocytes to MSC differentiation was also examined. Ter119^+^ nucleated bone marrow erythrocytes of different genotypes were isolated using magnetic microbeads conjugated with the Ter119 antibody. Cross co-culture was then performed to test the influence of erythroid progenitor cells on MSC differentiation. Interestingly, although both Ter119^+^ and Ter119^−^ cells of the Pten^+/flox^ genotype can largely improve the attenuated osteoblastic differentiation of Cre^+^Pten^+/flox^ MSCs (Fig. 5f, g, i), they cannot completely recover the extracellular matrix formation as the whole bone marrow does (Fig. 4Aa–f). Both Ter119^+^ and Ter119^−^ cells of the Cre^+^Pten^+/flox^ genotype significantly inhibited the calcium deposition of Pten^+/flox^ MSCs, and the Ter119^+^ subset functioned more powerfully (Fig. 5a, c, e). Similarly, Ter119^+^ and Ter119^−^ cells from Pten^+/flox^ mice ameliorated the augmented adipogenic differentiation of Cre^+^Pten^+/flox^ MSCs, with the former being more effective (Fig. 5f’, g’, i’). These data indicate that marrow erythropoiesis plays an important role in maintaining the normal differentiation activity of MSCs.

## Discussion

Hematopoiesis in bone marrow is maintained by niche cells including MSCs, and the indispensable role of MSCs in maintaining hematopoietic homeostasis has been fully elucidated [10–13, 15]. However, little is known about the mechanism by which hematopoietic cells reciprocally regulate niche cells. Addressing this question will be helpful for in vitro or ex vivo reproduction of their natural niche, which will promote their application in cell therapy. Here, we uncovered a close interrelationship between MSCs and hematopoietic cells, which may be exploited for the development of new therapeutic strategies.

During the development of mice and humans, MSCs and hematopoietic cells coexist in most of the hematopoietic tissues from the embryonic stage to adulthood [2, 3, 25–27], which also indicates an integrated relationship between the two types of cells. In vivo studies have shown that in the bone marrow of adult mice, MSCs are spatially associated with HSPCs to form a unique niche and they interact with each other [15]. In vitro studies have also indicated that both HSPCs and their progenies affect the differentiation activities of MSCs [16–18]. Together with previous reports, this study expands our understanding of the role of hematopoietic cells in regulating the function of MSCs. MSCs are the progenitor cells of osteoblasts, which are mainly responsible for bone formation. Therefore, the state of MSCs is closely related to bone homeostasis. Inhibition of osteogenesis was described in a recent leukemia mouse model, in which leukemic myeloid cells stimulate MSCs to proliferate and overproduce functionally altered osteoblasts by both direct cell–cell contact and secretion of soluble factors [28]. Here, we also identified aberrant secretion of cytokines known to regulate osteoblastic differentiation [28–30]. However, we found that hematopoietic cells balance the osteogenic and adipogenic differentiation of MSCs, mainly through direct contact with MSCs.

Pten dosage is closely related to the severity of various diseases [23, 24, 31], and Pten conditional loss in HSPCs leads to leukemia [19–21]. However, mice with Pten-specific half-deficient HSPCs were phenotypically normal and used as a control to study the role of Pten in hematopoiesis [19, 20]. Although hematopoiesis is normal in young mice, our data further demonstrated that the blood cell-specific haploinsufficiency of Pten leads to a progressive decrease in marrow erythropoiesis. In addition, Pten haploinsufficient hematopoietic cells weakened the osteoblastic differentiation potential and enhanced the adipogenic differentiation propensity of MSCs.

Both B and T lymphoid cells can affect MSC differentiation [32, 33]. However, the effect of erythroid cells in the bone marrow on MSCs is unknown. Erythropoietin can increase peripheral erythropoiesis and finally lead to bone loss; however, marrow erythropoiesis is normal and may concurrently impair B cell development, resulting in bone defects [33]. The erythroid lineage was found to be closely associated with bone, indicating that erythroid cells may contribute to bone homeostasis [34]. Our data showed that bone marrow erythroid progenitor cells could directly modulate the osteoblastic and adipogenic differentiation of MSCs in vitro*.* Erythropoiesis gradually becomes abnormal in mice with Pten haploinsufficiency in HSPCs, which may influence the differentiation activity of MSCs and finally lead to the progressive attenuation of bone formation in vivo.

The results indicate that hematopoietic cells function reciprocally as essential niche cells for MSCs, which would help to establish their natural niche to obtain cells of best quality. Because Tie2 is expressed in hemangioblasts, the common progenitor of endothelial and blood cells [35], Pten is also heterozygous null in endothelial cells of Cre^+^Pten^+/flox^ mice, which may be another reason for the altered differentiation activity of MSCs and the cause of abnormal bone development in vivo. The influence of endothelial cells on MSC function will be further explored in future studies. Our ongoing research showed that abnormal bone development caused by hematopoietic disease could be rescued by bone marrow transplantation, which provides direct evidence for blood-modulating osteogenesis. Thus, our data will be beneficial to the reproduction of natural niches in vitro for MSC proliferation and provide treatment strategies for bone diseases.

## Conclusions

Our results propose that hematopoietic cells function reciprocally as essential niche cells for MSCs. Progressive hematopoiesis abnormalities alter the biological characteristics of MSCs. Understanding the extrinsic features that function as niche for MSCs would help us to establish their natural niche to obtain cells of best quality and maximize their therapeutic benefit.

## Data Availability

All data generated and/or analyzed during this study are included in this published article.

## Abbreviations

MSCs: Mesenchymal stem cells
Pten: The phosphatase and tensin homolog deleted on chromosome 10
HSPCs: Hematopoietic stem/progenitor cellshematopoietic stem/progenitor cells
FBS: Fetal bovine serum
PE: Phycoerythrin
qPCR: Quantitative PCR
